# The prevention of contrast induced nephropathy by sarpogrelate in patients with chronic kidney disease: a study protocol for a prospective randomized controlled clinical trial

**DOI:** 10.1186/1745-6215-11-122

**Published:** 2010-12-20

**Authors:** Kyungil Park, Woo-Young Chung, Jae-Bin Seo, Sang-Hyun Kim, Joo-Hee Zo, Myung-A Kim, Young-Bae Park

**Affiliations:** 1 Division of Cardiology, Department of Internal Medicine, Seoul National University College of Medicine, Seoul, South Korea; 2 Cardiovascular Center, Seoul Metropolitan Boramae Medical Center, Seoul, South Korea; 3 Cardiovascular Center, Seoul National University Hospital, Seoul, South Korea

## Abstract

**Background:**

Contrast-induced nephropathy (CIN) is a serious clinical problem associated with increased morbidity and mortality, particularly in patients with chronic renal insufficiency. Although some agents including hydration with saline are being prescribed to prevent renal deterioration in these high risk patients, their efficacy is not clearly defined and debatable. Therefore additional prophylactic pretreatments are needed.

**Methods/Design:**

The present study aims to investigate differences in occurrence of CIN after sarpogrelate premedication in patients with chronic kidney disease (CKD). 268 participants, aged 20-85 years with a clinical diagnosis of CKD will be recruited. They will be randomly allocated to one of two conditions: (i) routine treatment without sarpogrelate, and (ii) routine treatment with sarpogrelate (a fixed-flexible dose of 300 mg/day). The primary outcome is the occurrence of CIN during 4 weeks after receiving contrast agent.

**Discussion:**

As of May 2010, there were no registered trials evaluating the therapeutic potentials of sarpogrelate in preventing for CIN. If sarpogrelate decreases the worsening of renal function and occurrence of CIN, it will provide a safe, easy and inexpensive treatment option.

**Trial registration:**

NCT01165567

## Background

Contrast-induced nephropathy (CIN) is a common form of hospital-acquired acute renal failure (ARF) after coronary angiography (CAG) and percutaneous coronary intervention (PCI) and is associated with prolonged hospitalization and adverse clinical outcomes [1,2]. Patients undergoing PCI have a higher mortality rate if the nephropathy develops [3]. Although its incidence is low in patients with normal renal function, it can be much higher in those with renal insufficiency at baseline [4,5]. Adequate prophylaxis is needed to reduce the higher morbidity and mortality associated with CIN in high-risk patients. A variety of therapeutic interventions, including saline hydration, diuretics, mannitol, calcium channel antagonists, theophylline, endothelin receptor antagonists, and dopamine, have been used in an attempt to prevent CIN [6-9]. Hydration with normal saline solution is the most widely accepted preventive intervention [9,10]. But the nephropathy occurred in 20~30% who received the recommend treatment [11,12], it means current treatments are not enough and the optimal strategy to prevent CIN has not been established.

Although the pathogenesis of this condition is not fully understood, it is most likely the result of renal ischemia and direct toxicity to tubular epithelial cells [13,14]. After contrast is injected, renal blood flow transiently increases and then decreases over a longer time, suggesting that renal ischemia is a major factor in the pathogenesis of CIN [13]. Contrast agents reduce the oxygen tension in both the cortex and the medulla [14]. Many studies suggest that contrast agents are directly toxic to kidney cells, causing proximal cell vacuolization, interstitial inflammation, cellular necrosis, and enzymuria [15].

**Figure 1:**
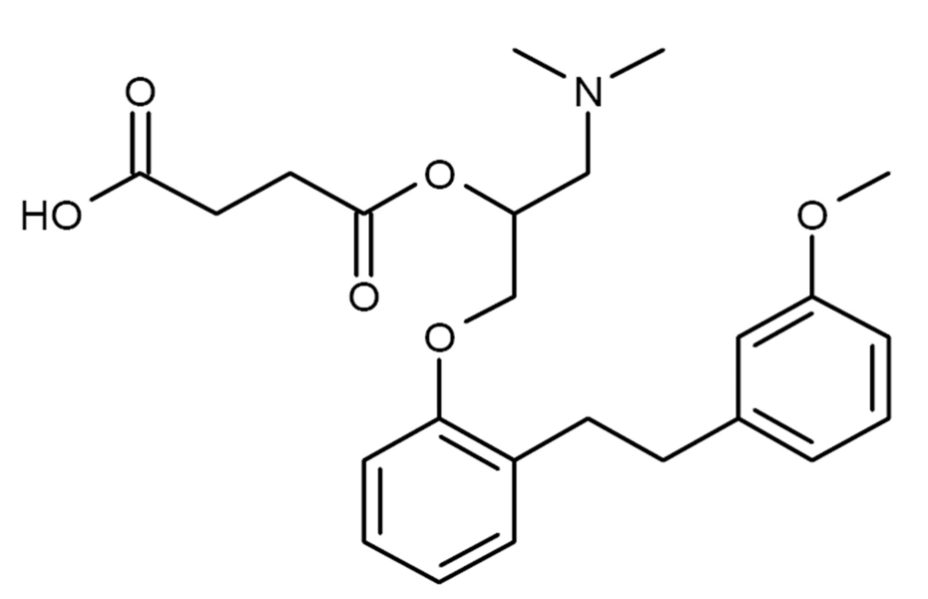
**Structure of sarpogrelate**.

Sarpogrelate is a selective 5-hydroxytryptamine receptor subtype 2A (5-HT_2A_) antagonist that inhibits responses to 5-HT mediated by 5-HT_2A _receptors such as platelet aggregation, vasoconstriction, and vascular smooth muscle proliferation [16-18]. Its chemical structure is illustrated in Figure 1. Several studies demonstrated that sarpogrelate is efficacious treatment in thrombosis, coronary artery spasm, atherosclerosis, restenosis, peripheral artery disease, diabetes and kidney disease [16,17,19-23]. Especially small clinical trial had shown that sarpogrelate has a renal protective potential [23,24]. A pilot study demonstrated that sarpogrelate improved the symptoms and signs of diabetic nephropathy and neuropathy in three of eight patients with Type 2 diabetes and mild nephropathy [25]. Sarpogrelate also reduced the urinary and plasma levels of thromboxane A_2 _and this may be associated with the reduced albumin excretion [26]. Another study demonstrated that sarpogrelate reduced albuminuria in diabetic patients with early-stage diabetic nephropathy within 3 months [27]. Therefore, we might hypothesize that sarpogrelate would have shown positive results in prophylaxis of CIN.

The primary hypothesis to be tested is whether sarpogrelate significantly reduce the occurrence of CIN during 4 weeks follow-up period, compared with the control group.

## Methods/Design

The study is designed as a non-blinded, prospective, randomized controlled clinical trial to evaluate efficacy and safety of sarpogrelate in CKD patients undergoing CAG and PCI. After a baseline measurement, participants were randomly allocated into the treatment group (routine treatment with sarpogrelate) or the control group (routine treatment without sarpogrelate). In this study, routine treatment is defined as saline hydration and benefit of sarpogrelate is assessed as an adjunct to saline hydration for the prevention of CIN following diagnostic CAG or PCI

Study approval was given by the Ethics Committee/Institutional Review Board of Seoul National University Boramae Medical Center.

### Participants

All patients with CKD scheduled for CAG will be eligible, assuming their ability to understand the character and individual consequences of participation as well as giving written informed consent. CKD is defined as estimated glomerular filtration rate (eGFR) <60 mL/min per 1.73 m^2 ^using modification of diet in renal disease (MDRD) formula or serum creatinine (Cr) > 1.5 mg/dL.

The MDRD formula was defined in the following way. Where the Cr concentration is in mg/dL:

$$\text{eGFR}=\text{186}\times {\text{serum Cr}}^{-1.154}\times {\text{Age}}^{-0.203}\times \left(0.\text{742 if female}\right)$$

Exclusion criteria are age less than 20 years or more than 85 years, liver cirrhosis greater than or equal to Child class B, decreased serum platelet level (< 100,000/uL), patients who received or are schedule to receive percutaneous renal intervention, currently are taking anticoagulation drugs, unable to give informed consent. Patients in shock, maintaining hemodialysis, hemofiltration, peritoneal dialysis will be excluded.

### Intervention

After a baseline measurement, all patients receive 0.9% saline or 0.45% saline intravenously at 150 ml/hour at least for 6 hours before and 6~8 hours after receiving contrast agent. Patients in the treatment group receive sarpogrelate 300 mg per day for 24 hours before exposure to contrast agent. Sarpogrelate will be given for 4 weeks.

**Figure 2:**
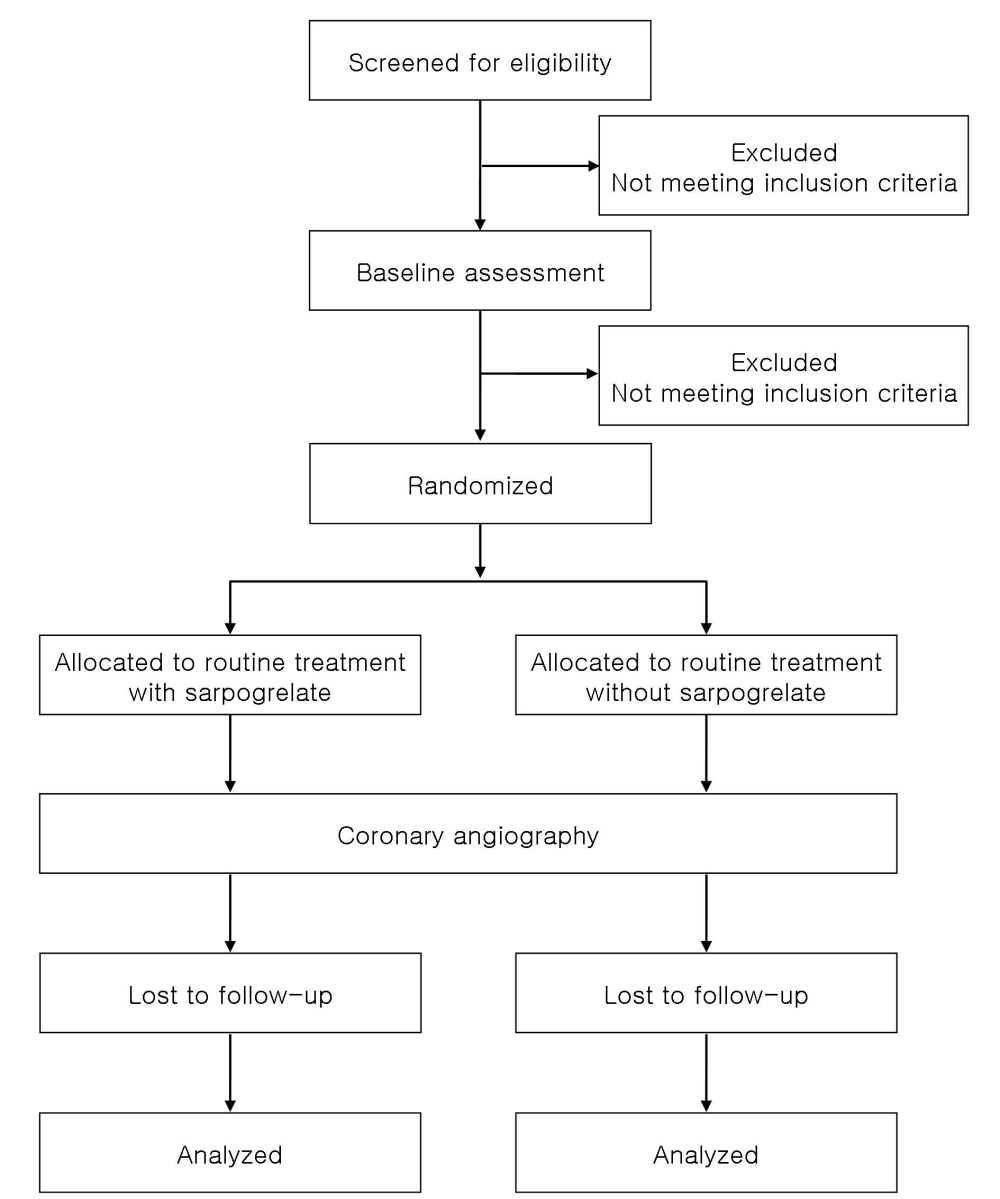
**Study design**.

Recruitment of participants started in April 2010. The process of the trial conduct is illustrated in Figure 2. No additional risks for study patients are expected, since all treatment is carried out within established standard methods of CAG or intervention.

### Primary Outcome Measurement

The primary outcome of the study is the incidence of CIN, defined as either a greater than 25 percent increase of serum Cr or an absolute increase in serum Cr of 0.5 mg/dL within 48 hours after using contrast agent [28,29]. Clinical endpoint measurements are conducted in-hospital and at 4 weeks. Cr concentration is measured at admission, every day for the next two days after contrast exposure, and at 4 weeks.

### Secondary Outcomes Measurement

Secondary outcomes are occurrence of CIN, performance of hemodialysis or hemofiltration and bleeding events at 4 weeks.

### Adverse effects

Analysis of safety related data is performed with respect to frequency of serious adverse events, frequency of serious adverse events stratified by causality and frequency of morbidity in both treatment groups.

At each assessment patients are assessed to report bleeding. Patients who experienced a bleeding event are further classified based on bleeding severity according to the Global Use of Strategies to Open Occluded Coronary Arteries (GUSTO) bleeding criteria [30]. Severe bleeding is defined as any intracranial hemorrhage or bleeding that causes hemodynamic compromise and requires intervention. Moderate bleeding is defined as bleeding requiring transfusion but does not result in hemodynamic compromise. Mild bleeding is defined as bleeding that does not meet criteria for either severe or moderate bleeding. Serious adverse events (SAE) have to be reported by the attending physician to the principal investigator within 24 hours after the SAE becomes known.

### Withdrawals

Patients are free to withdraw trial participation at their own request at any time and without giving reasons for their decision. Moreover, the primary investigator can withdraw study patients, if continuation of the trial would be detrimental to the patient's well being. Withdrawals will be documented in the Case report form (CRF) and in the patient's medical records and all ongoing SAE have to be followed up.

### Sample size

The sample size calculation is based on the primary outcome and the primary analysis for the intention-to-treat population. The sample size is calculated using the study's primary objective to detect a 50% difference of CIN occurrence between routine treatment with sarpogrelate and without sarpogrelate in a power of 80% to demonstrate difference. We assumed that the expected rate of CIN would be 20% in the control group [11]. We adjust the sample size for an estimated follow-up loss rate of 10%, a two-sided level of significance α = 5% and a power of 1-β = 80%, which result in 134 patients in each group is required to detect this difference with a two-sided Student's t- test. A total of 268 patients will be randomized and included in the analysis.

### Randomization

Random assignments are generated using Excel spreadsheet software (Microsoft Corporation, Redmont, USA). Eligible patients are randomly assigned in a 1:1 ratio to receive routine treatment with sarpogrelate or routine treatment without sarpogrelate. Randomization is performed by a personnel not involved in the study and kept concealed.

### Statistical analysis

The statistical analyses are performed on an intention-to-treat basis. The two-sided null-hypothesis for the primary outcome measure states that sarpogrelate has the potential effect on preventing CIN in CKD patients undergoing CAG or PCI. Descriptive statistics will be calculated according to the scale level of the variables. The Kaplan- Meier method is used to plot the time to the first episode of CIN, performance of hemodialysis or hemofiltration and bleeding events after CAG or PCI.

Continuous data will be analyzed using Student's t-test. The chi-square test is used for categorical variables. A value of p < 0.05 is considered statistically significant. Efficacy analyses was performed on the full analysis set, which consist of patients who received at least 1 dose of study medication. Patients who take the medication at least once are analyzed for safety. Graphical methods including scatter plots and boxplots will be used to visualize the findings of the trial.

The safety analysis includes calculation of frequencies and rates of complications and serious adverse events reported in the two groups. All analyses will be done using SPSS Version 16.0 or higher.

### Approval

This study follows the Helsinki Declaration's principles, meaning that all patients sign a written informed consent stating that participation is voluntary and that participation can be withdrawn at any time, without any negative consequences concerning their current or future medical treatment. This study protocol was approved by the institutional review board of Seoul National University Boramae Medical Center.

## Discussion

Sarpogrelate has shown promising results in animal studies and clinical trials. Recent studies show that this drug has renal protective effect, however, its effects is still not fully understood.

In this study, we examine the hypothesis that sarpogrelate has the prophylactic potential in CIN based on the results of previous trials. The present study is small and short-term clinical trial but the first trial investigating whether sarpogrelate has an effect on preventing CIN in CKD patients undergoing CAG or PCI.

If the potential effects of sarpogrelate on these events are proved, the result of present study may provide an inexpensive, safe, practical, and simple method for preventing CIN other than saline hydration.

## List of abbreviations

CIN: Contrast-induced nephropathy; CKD: Chronic kidney disease; CRF: Case Report Form; GUSTO: Global Use of Strategies to Open Occluded Coronary Arteries; PCI: Percutaneous coronary intervention.

## Competing interests

The authors declare that they have no competing interests.

## Authors' contributions

WYC is the Principle Investigator for the study, contributed to the study design and to drafting and revising the manuscript. KP made significant contributions to concept of the study, drafting and reviewing manuscript. All authors read and approved the final manuscript.
